# BNP-Derived Uric Acid Composite Indices for the Evaluation of Dyspnea in Third-Trimester Pregnancy: A Prospective Emergency Department Study

**DOI:** 10.3390/jcm15145456

**Published:** 2026-07-12

**Authors:** Tarık Acar, Birsen Ertekin, Azmi Eyiol, Fatih İkiz, Ahmet Yilmaz, Rukiye Ozcelik Tepe

**Affiliations:** 1 Department of Emergency, Beyhekim Training and Research Hospital, Selcuklu 42130, Konya, Türkiye; biceacil@hotmail.com (B.E.); sultanmehmet01@hotmail.com (F.İ.); 2 Department of Cardiology, Beyhekim Training and Research Hospital, Selcuklu 42130, Konya, Türkiye; azmieyiol@yahoo.com; 3 Department of Cardiology, Karamanoglu Mehmetbey University Faculty of Medicine, Karaman 70100, Türkiye; dr.ahmetyilmaz@gmail.com; 4 Department of Obstetric Gynecology, Beyhekim Training and Research Hospital, Selcuklu 42130, Konya, Türkiye; drrozcelik@gmail.com

**Keywords:** pregnancy, dyspnea, echocardiography, B-type natriuretic peptide, D-dimer, uric acid, emergency department

## Abstract

**Background:** Dyspnea is common in pregnancy and difficult to assess in the emergency department (ED), as it may reflect physiological adaptation or cardiopulmonary pathology. We evaluated whether novel BNP-derived uric acid composite indices aid the assessment of dyspnea in late pregnancy; to our knowledge, this is the first study of such indices in this setting. **Methods:** In this prospective observational study, third-trimester pregnant women (≥27 weeks) presenting to the ED with dyspnea were compared with age-matched asymptomatic pregnant controls. All underwent laboratory testing (including BNP, D-dimer, and uric acid) and standardised transthoracic echocardiography. Three composite indices were calculated (BNP/Albumin, BNP/Uric acid, and BNP × Uric acid), and diagnostic performance was assessed by receiver operating characteristic (ROC) analysis. **Results:** A total of 215 women were included (100 dyspnea, 115 controls). The dyspnea group had significantly higher D-dimer, BNP, uric acid, and all three composite indices (all *p* < 0.001), together with higher systolic pulmonary artery pressure (sPAP), right atrial and ventricular diameters, and lateral E/e′. Uric acid (AUC 0.998), the BNP × Uric acid product (AUC 0.846), and sPAP (AUC 0.836) showed the strongest discrimination; the BNP × Uric acid product outperformed BNP, D-dimer, and BNP/Albumin individually but did not surpass uric acid alone. **Conclusions:** Dyspneic third-trimester women show subtle but significant cardiopulmonary changes despite values that largely remain within normal ranges. These changes distinguished symptomatic from asymptomatic pregnancy but remained largely within normal ranges and do not by themselves identify acute cardiopulmonary disease; uric acid alone was the strongest discriminator, and the composite indices are proposed only as hypothesis-generating tools requiring external validation, ideally against dyspnoeic pregnant women with confirmed pathology.

## 1. Introduction

Dyspnoea is one of the most frequent reasons for pregnant women to visit the emergency department (ED). It is commonly observed during the final trimester of pregnancy and may be linked to various underlying conditions [1,2]. Healthy pregnant women experience changes in their cardiopulmonary system due to increased volume load. Understanding these changes is crucial for recognising dyspnoea [3]. Cardiopulmonary diseases are major causes of maternal mortality and morbidity. It is important to distinguish whether dyspnoea during pregnancy is physiological or pathological [4]. Pregnancy is characterised by significant haemodynamic changes, and it is a period of increased cardiovascular risk for women. Therefore, pregnant women presenting with symptoms such as dyspnoea, chest pain, and tachycardia should undergo a more thorough assessment, even if they have no pre-existing cardiovascular problems [5]. Currently, echocardiography is a non-invasive and reliable diagnostic tool that allows for the assessment of both the reversible physiological cardiac remodelling of pregnancy and the evaluation of suspected heart disease [6]. Physiological changes during pregnancy can affect the cardiovascular system, leading to dyspnoea. Therefore, echocardiography is recommended to investigate the aetiology of dyspnoea [4,7]. A recent study has demonstrated that during a normal pregnancy, cardiac chamber dimensions in both systole and diastole progressively increase throughout the trimester and return to pre-pregnancy size by the sixth week postpartum [8]. In a study by McGourty M et al. involving 478 pregnant patients, abnormal.

B-type natriuretic peptide (BNP) is released from the myocardium in response to atrial and ventricular wall stretch and is widely used to differentiate heart failure from other causes of dyspnoea, although studies in pregnancy have shown conflicting results [5]. For example, in one study, BNP levels were found to be similar between pregnant women with and without congenital heart disease [9]. Another study found that high BNP levels help diagnose heart failure in pregnant and postpartum women [10].

The risk of venous thromboembolism (VTE) is higher in pregnant women compared to non-pregnant women. In developed countries, pulmonary embolism (PE) is the main cause of maternal mortality. The effectiveness of D-dimer levels in ruling out VTE in pregnant women remains uncertain because these levels are consistently elevated during pregnancy [11]. A recent study has shown that D-dimer ≥ 3.70 mg/L were independently associated with VTE during the puerperium period [12].

Beyond BNP and D-dimer, serum uric acid has increasingly been recognised as a marker of cardiovascular and metabolic stress. Elevated uric acid reflects oxidative stress, endothelial dysfunction, and impaired renal handling of urate, and has been linked to adverse outcomes in heart failure independent of natriuretic peptide levels [13]. Composite indices combining natriuretic peptides with markers of nutritional or metabolic status—such as the blood urea nitrogen/albumin ratio and the uric acid/albumin ratio—have recently been proposed as integrative prognostic tools in cardiovascular and critical care populations, on the rationale that combining a marker of myocardial stretch with a marker of systemic stress may capture pathophysiological information that neither marker conveys alone [14]. To date, no study has examined whether similar BNP-derived composite indices—specifically BNP normalised to albumin, BNP normalised to uric acid, and the BNP × uric acid product—carry diagnostic value in pregnant women presenting with dyspnoea.

From a pathophysiological perspective, dyspnea during late pregnancy may reflect the interplay between physiological hemodynamic adaptation, subtle myocardial wall stress, endothelial dysfunction, and oxidative–metabolic alterations. While BNP primarily reflects myocardial stretch and intracardiac pressure changes, serum uric acid has been associated with oxidative stress, endothelial dysfunction, inflammation, and impaired vascular adaptation. Therefore, combining biomarkers that represent different biological pathways may provide a more comprehensive assessment than isolated markers alone. In recent years, composite biomarker approaches have attracted increasing interest because they may better capture the complex pathophysiological mechanisms underlying cardiovascular and systemic disorders.

Therefore, we aimed to evaluate conventional biomarkers and echocardiographic findings in third-trimester pregnant women presenting to the emergency department with dyspnea and, for the first time, to investigate the diagnostic performance of novel BNP-derived uric acid composite indices, including the BNP/albumin ratio, BNP/uric acid ratio, and BNP × uric acid product. In addition, asymptomatic third-trimester pregnant women were included as controls to establish reference biomarker and echocardiographic profiles and to identify subtle hemodynamic alterations associated with dyspnea. To the best of our knowledge, this is the first study to evaluate BNP-derived uric acid composite indices in this specific clinical setting.

## 2. Materials and Methods

### 2.1. Study Design and Patient Population

Between 1 October 2023 and 1 May 2025, pregnant patients in their third trimester (≥27 weeks’ gestation) who presented to the ED of a teaching and research hospital were evaluated prospectively. Eligibility criteria included patients aged over 18 who provided informed consent and had comprehensive clinical and laboratory information accessible through the hospital’s record system.

Upon presentation to the ED, the following patient data was collected and recorded on a pre-prepared form: age, body mass index (BMI), gravidity, gestational week, mean arterial pressure (MAP), pulse pressure, white blood cell (WBC) count, haemoglobin (Hb), platelet count (PLT), troponin I, D-dimer, B-type natriuretic peptide (BNP), C-reactive protein (CRP), albumin, uric acid, thyroid-stimulating hormone (TSH), glucose, creatinine, triglyceride levels, and Doppler echocardiography findings interpreted by a cardiologist. A control group was also established, consisting of healthy, non-dyspnoeic, volunteer pregnant patients in their third trimester, who were of a similar age. The same laboratory tests were performed on the control group patients, and they also underwent echocardiography. All echocardiographic evaluations were performed by the same cardiologist using a standard protocol. Parasternal long axis, short axis, apical four-chamber, and subcostal views were routinely assessed in accordance with ASE (American Society of Echocardiography) guidelines [15]. All echocardiographic examinations were performed by the same experienced cardiologist, who was unaware of the calculated composite biomarker indices at the time of image acquisition and interpretation. Measurements were obtained according to current American Society of Echocardiography recommendations to minimise interobserver variability.

Patients excluded from the study were those under 18, with obesity, multiple pregnancies, or acute/chronic conditions such as haematological, cardiac, valvular, hepatic, or renal disease. Other exclusions included hypertension, asthma, chronic obstructive pulmonary disease (COPD), PE, diabetes mellitus, cancer, pre-eclampsia/eclampsia, active pulmonary infection, recent poisoning or trauma, immunosuppressed individuals, and those who did not provide informed consent, had a gestational age of ≤27 weeks, or whose information was inaccessible from electronic medical records. All laboratory and echocardiography findings were compared between the patient and control groups. The study received approval from the Necmettin Erbakan University Faculty of Medicine Ethics Committee on 15 September 2023, with the reference number 2023/4538(15716). Prior to their inclusion in the study, both patients and volunteers were informed about the study and provided their written consent.

In this study, dyspnoea was recorded as the patient’s self-reported chief complaint of breathlessness at emergency department presentation; owing to the acute clinical setting and patient flow, no formal dyspnoea severity instrument (such as the NYHA, modified Borg, or mMRC scale) was applied to grade its intensity. Because all major organic causes of dyspnoea were removed by the exclusion criteria above—including asthma, chronic obstructive pulmonary disease, pre-eclampsia, hypertension, pulmonary embolism, active pulmonary infection, and obesity—the dyspnoea group represents women with physiological or otherwise unexplained gestational dyspnoea rather than an acute cardiopulmonary emergency. Patients were discharged once acute conditions had been excluded and were not followed for a definitive etiological diagnosis; accordingly, the present analysis characterises biomarker and echocardiographic differences between symptomatic and asymptomatic third-trimester pregnancy and is not intended as a diagnostic triage tool for acute disease.

The natriuretic peptide measured in this study was B-type natriuretic peptide (BNP); N-terminal pro-B-type natriuretic peptide (NT-proBNP) was not assayed, and all composite indices were derived exclusively from measured BNP. NT-proBNP appears in this manuscript only when prior studies that employed it are discussed. Beyond routinely documented vital signs (blood pressure and heart rate, which were within normal limits in both groups), additional acute-presentation variable—peripheral oxygen saturation, respiratory rate, chest pain, syncope, suspected pulmonary-embolism work-up, and emergency-department disposition—were not systematically recorded, nor was a formal dyspnoea-severity instrument (NYHA, modified Borg, or mMRC) applied.

The primary objective of the study was to evaluate the diagnostic performance of novel BNP-derived uric acid composite indices in distinguishing third-trimester pregnant women presenting to the emergency department with dyspnea from asymptomatic age-matched pregnant controls.

### 2.2. Hematologic and Biochemical Analysis

Complete blood count parameters were measured using a Mindray auto haematology analyser BC-6800 (Mindray, Shenzhen, China). Biochemical parameters, including serum uric acid and albumin, were obtained using a Mindray chemistry analyser BS-2000M (Mindray, Shenzhen, China). Echocardiography imaging was performed with a Philips EPIQ CVx device (Philips, Glemsford, UK).

### 2.3. Composite BNP-Derived Indices

To explore whether normalising or combining BNP with markers of nutritional and metabolic stress would improve diagnostic discrimination, three composite indices were calculated for each participant from the raw laboratory values: (i) the BNP/Albumin ratio, calculated as serum BNP (pg/mL) divided by serum albumin (g/L); (ii) the BNP/Uric acid ratio, calculated as serum BNP (pg/mL) divided by serum uric acid (mg/dL); and (iii) the BNP × Uric acid product, calculated as serum BNP (pg/mL) multiplied by serum uric acid (mg/dL). These indices were treated as continuous variables and were compared between groups using the same statistical approach applied to the other laboratory parameters, and their diagnostic performance was subsequently assessed with ROC analysis. These composite indices were designed to integrate information related to myocardial wall stress and metabolic–oxidative status and were analysed as continuous variables throughout the study.

### 2.4. Statistical Analysis

The analysis focused on assessing the discriminative performance of conventional biomarkers, echocardiographic parameters, and novel BNP-derived composite indices. Receiver operating characteristic (ROC) analysis was performed to evaluate diagnostic accuracy, and optimal cut-off values were determined using the Youden index. Statistical analyses in the study were performed using Python (version 3.12) with the SciPy (v1.17), NumPy (v2.4), pandas, and scikit-learn (v1.8) libraries. The Kolmogorov–Smirnov test, histogram analyses, and kurtosis/skewness data were used to evaluate the normality assumptions of continuous variables. Continuous variables were expressed as the median with the 25th–75th percentile (interquartile range, IQR) or mean ± SD according to their distribution. Qualitative data are expressed as frequency and percentage (%). Homogeneity assumptions of normally distributed parameters were checked with Levene’s test. Relationships between two independent groups were examined with the Mann–Whitney U test or the independent *t*-test, as appropriate. Confounders (age, BMI, MAP, and gestational week) were controlled with ANCOVA analysis, modelled via ordinary least-squares regression comparing the full model (group plus covariates) against the reduced model (covariates only); the corresponding F-statistic, *p*-value, and partial eta-squared (η^2^p) were derived from the resulting sum-of-squares decomposition. Relationships between qualitative parameters were carried out with Pearson’s chi-square analysis. Correlations between continuous variables were carried out with Spearman’s correlation analysis. ROC curve analysis was used to determine the discriminative performance of laboratory and echocardiographic parameters; the area under the curve (AUC), 95% confidence interval (Hanley-McNeil method), optimal cut-off (Youden index), sensitivity, and specificity were calculated for each parameter. Pairwise comparisons between ROC curves—in particular uric acid versus each BNP-derived composite index, and the BNP × Uric acid product versus BNP and D-dimer—were performed using the DeLong test for correlated areas under the curve [16]. In the entire study, the type-I error rate was taken as 5% (α = 0.05), and a *p* < 0.05 level was considered as the significance threshold.

## 3. Results

**Figure 1:**
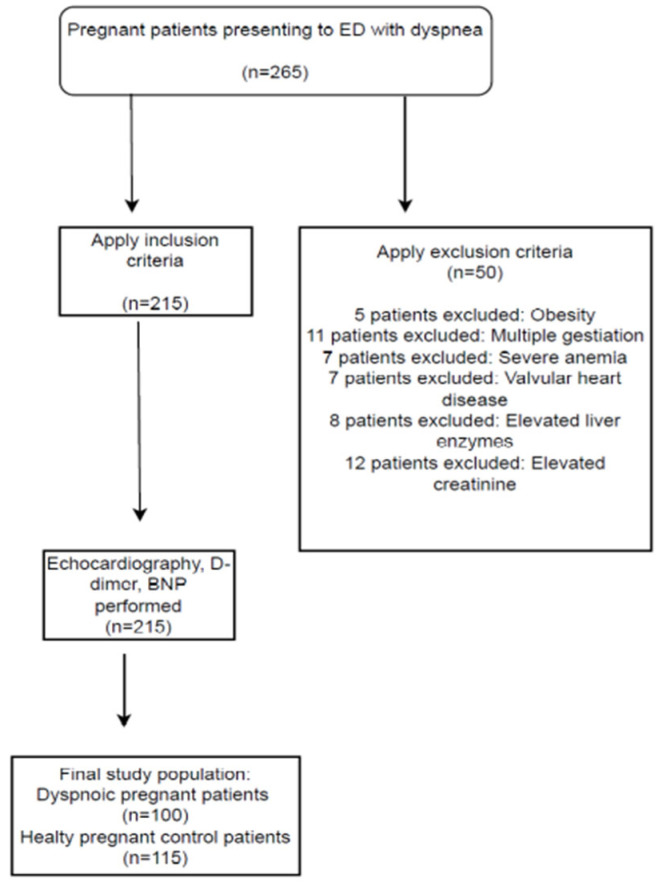
Patient flow diagram showing enrolment, exclusions, and group allocation. BNP, B-type natriuretic peptide; ED, emergency department.

**Figure 2:**
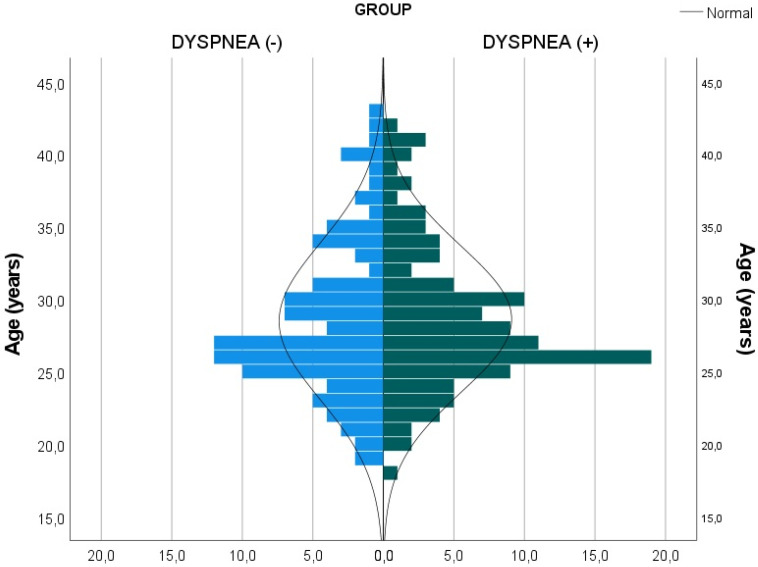
Distribution of age within groups.

Figure 1 presents the patient flow diagram showing enrollment, exclusions, and group allocation. A comparison of the demographic and laboratory parameters between the patient and control groups is presented in Table 1. Out of a total of 215 patients, 115 (53.5%) constituted the control group, and 100 (46.5%) formed the patient group. There was no statistically significant difference in age between the groups (*p* = 0.706), and the distribution graph is shown in Figure 2. When both groups were compared in terms of BMI, gravidity, gestational week, MAP, pulse pressure, glucose, creatinine, TSH, troponin I, and albumin, no significant difference was observed (*p* > 0.05 for all). In the patient group, D-dimer, BNP, uric acid, triglycerides, CRP and WBC count were found to be significantly higher (*p* < 0.05 for all).

Importantly, all three novel BNP-derived composite indices were significantly higher in the patient group than in the control group: the BNP/Albumin ratio (0.9 ± 0.5 vs. 0.6 ± 0.2; *p* < 0.001), the BNP/Uric acid ratio (5.6 ± 2.7 vs. 4.0 ± 1.4; *p* < 0.001), and the BNP × Uric acid product (196.3 ± 97.0 vs. 97.7 ± 33.0; *p* < 0.001). These findings indicate that the dyspnoeic group not only showed elevated BNP in isolation, but also a disproportionate elevation in BNP relative to both nutritional (albumin) and metabolic-stress (uric acid) markers. Notably, among the novel composite indices, the BNP × uric acid product exhibited the largest absolute difference between groups, suggesting that simultaneous assessment of myocardial and metabolic stress may better characterise the physiological alterations associated with dyspnea during late pregnancy.

Table 2 provides a comparison of the echocardiographic findings between the patient and control groups. No statistically significant differences were observed when comparing both groups for left ventricular end-diastolic diameter (LVEDd), left ventricular end-systolic diameter (LVESd), interventricular septum thickness (IVSd), left ventricular posterior wall thickness (LVPWd), left atrial diameter, aortic root diameter, aortic systolic diameter, aortic diastolic diameter, mitral E wave velocity, mitral A wave velocity, septal a’ wave velocity, and E/A ratio (*p* > 0.05 for all). In the patient group, the right atrial diameter, right ventricular diameter, systolic pulmonary artery pressure (sPAP), TAPSE, septal E/e’, and lateral E/e’ were found to be significantly different from controls. Conversely, septal e’ wave velocity, lateral e’ wave velocity, and lateral a’ wave velocity were detected as lower (*p* < 0.05 for all).

Correction for confounding factors (age, BMI, MAP, and gestational week) was applied during the comparison of laboratory parameters between the sample groups, and it was confirmed that potential secondary variables did not account for the observed group differences (Table 3). Notably, uric acid retained an exceptionally large effect size after adjustment (F = 1221.8, *p* < 0.001, η^2^p = 0.854), and the BNP × Uric acid product retained a substantially larger adjusted effect size (η^2^p = 0.326) than BNP alone (η^2^p = 0.239), D-dimer (η^2^p = 0.203), or the BNP/Uric acid ratio (η^2^p = 0.122), suggesting that the multiplicative combination of BNP and uric acid captures group-related variance beyond either marker individually. Importantly, the superior effect size observed for the BNP × uric acid product compared with BNP alone suggests that combining markers representing distinct pathophysiological domains may capture additional group-related variance beyond isolated biomarkers.

The ROC analysis of the parameters is presented in Table 4. Among the laboratory parameters, uric acid demonstrated near-perfect discrimination (AUC 0.998, 95% CI 0.991–1.000), with a sensitivity of 99.0% and specificity of 97.4% at a cut-off of ≥5.6 mg/dL. Of the conventional markers, BNP showed the highest AUC (0.769; sensitivity 65.0%, specificity 82.6%; cut-off ≥25.3 pg/mL), followed by D-dimer (AUC 0.754; sensitivity 54.0%, specificity 95.7%; cut-off ≥1090 µg/L). Among the three novel composite indices, the BNP × Uric acid product achieved the highest discriminative performance (AUC 0.846, 95% CI 0.792–0.900; sensitivity 70.0%, specificity 91.3%; cut-off ≥138), exceeding the AUC of BNP, D-dimer, triglyceride, CRP, and WBC individually. The BNP/Albumin ratio performed comparably to BNP alone (AUC 0.765), while the BNP/Uric acid ratio showed the lowest AUC among the composite indices (0.671), likely reflecting the attenuating effect of dividing by an already highly discriminative denominator. Overall, the BNP × uric acid product demonstrated the highest discriminative performance among the evaluated composite indices, whereas the BNP/uric acid ratio showed comparatively lower performance, suggesting that the multiplicative interaction between BNP and uric acid may better reflect the combined effects of myocardial wall stress and metabolic–oxidative burden.

In direct pairwise ROC comparisons (DeLong test), uric acid alone remained the single strongest discriminator and was superior to the BNP × Uric acid product (*p* < 0.001); the BNP × Uric acid product was, in turn, compared with BNP (*p* < 0.001) and with D-dimer (*p* = 0.021). It should therefore be emphasised that, although the BNP × Uric acid product exceeded BNP, D-dimer, and BNP/Albumin, it did not surpass uric acid measured alone.

**Figure 3:**
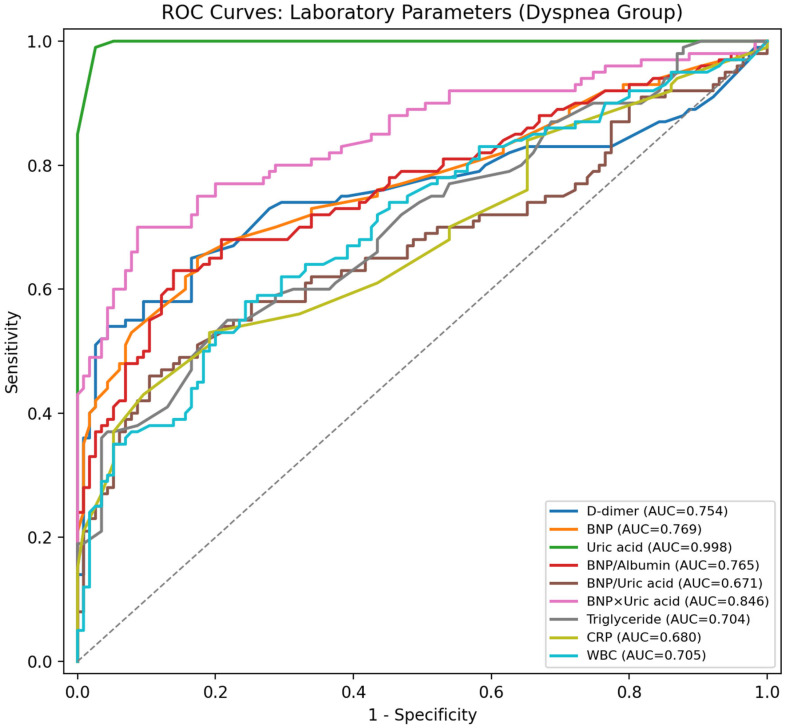
ROC analysis graph (laboratory parameters, including the novel BNP-derived composite indices) performed for the dyspnea group. BNP: brain natriuretic peptide, CRP: C-reactive protein, WBC: white blood cell, UA: uric acid.

**Figure 4:**
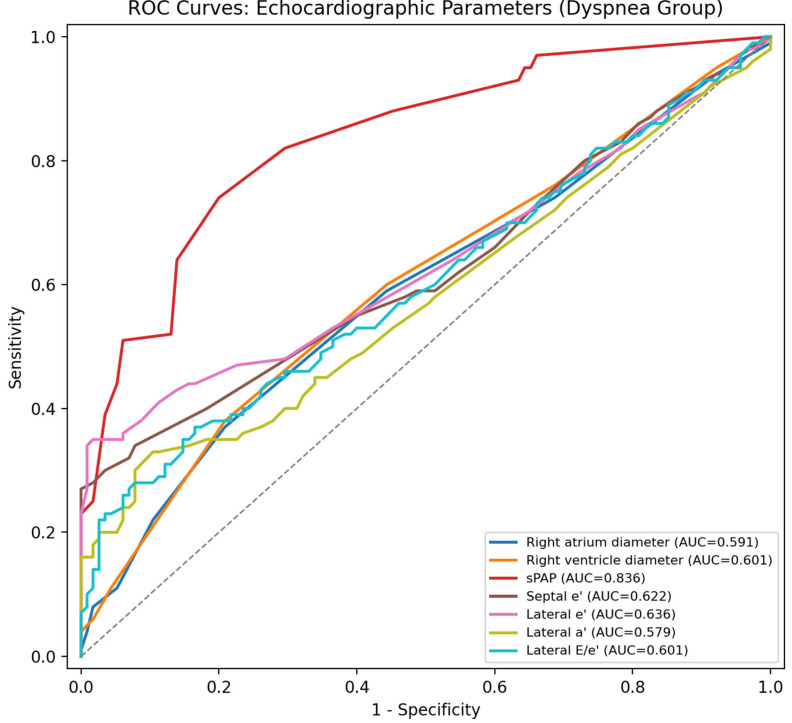
ROC analysis graph (echocardiography parameters) performed for the dyspnea group.

Among the echocardiographic findings, sPAP demonstrated the highest AUC value (0.836), while lateral a′ wave velocity had the lowest value (AUC 0.579). The ROC analysis graphs for both laboratory parameters (including the novel composite indices) and echocardiographic findings are presented in Figure 3 and Figure 4.

### Correlation Analyses

**Figure 5:**
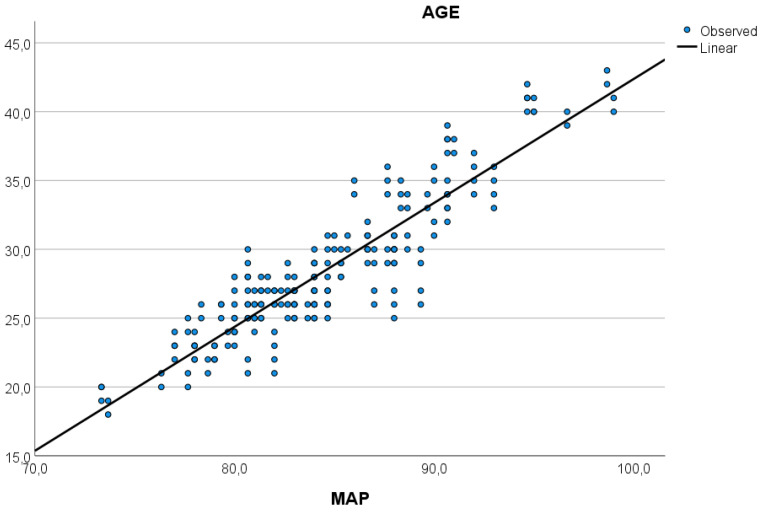
Strong positive correlation between age and MAP.

**Figure 6:**
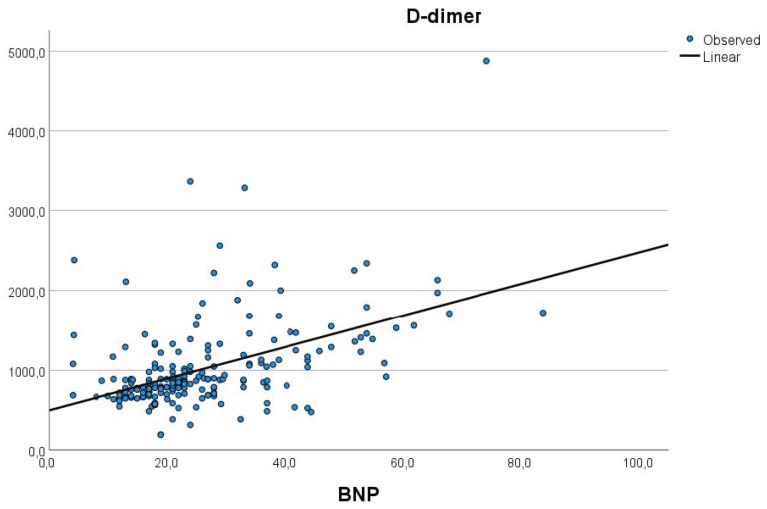
Moderate positive correlation between D-dimer and BNP.

Table 5 displays the correlation between age, BMI, MAP, and laboratory parameters, including the novel BNP-derived indices. A strong positive correlation was observed between age and MAP (Figure 5; rho = 0.879, *p* < 0.001). A moderate positive correlation was found between D-dimer and BNP (Figure 6; rho = 0.481, *p* < 0.001). All three composite indices correlated strongly with raw BNP (rho = 0.987, 0.971, and 0.979 for BNP/Albumin, BNP/Uric acid, and BNP × Uric acid, respectively; *p* < 0.001 for all), as expected given their shared BNP component, but the BNP × Uric acid product additionally showed a moderate-to-strong correlation with uric acid itself (rho = 0.561, *p* < 0.001) and with D-dimer (rho = 0.492, *p* < 0.001), the strongest D-dimer correlation among all laboratory markers tested.

Table 6 presents the correlation between age, BMI, MAP, D-dimer, BNP, uric acid, the BNP × Uric acid product, and echocardiographic parameters. A weak-to-moderate positive correlation was found between sPAP and BNP, D-dimer, and uric acid (rho = 0.398, 0.201, and 0.508, respectively; *p* < 0.01 for all). The BNP × Uric acid product showed a stronger correlation with sPAP (rho = 0.466, *p* < 0.001) than BNP alone, and a moderate correlation with right atrial and right ventricular diameters (rho = 0.206 and 0.213, respectively; *p* < 0.01 for both), surpassing the corresponding correlations for D-dimer and approaching those for BNP. TAPSE correlated negatively with uric acid (rho = −0.268, *p* < 0.01) and, more modestly, with the BNP × Uric acid product (rho = −0.135), consistent with a relationship between higher metabolic-stress biomarker levels and reduced right ventricular systolic function indices. Furthermore, the BNP × uric acid product showed stronger associations with sPAP and right heart dimensions than D-dimer and correlations broadly comparable to those observed with BNP, supporting a potential link between combined biomarker burden and subtle hemodynamic alterations in dyspneic pregnant women.

## 4. Discussion

Dyspnoea, regarded as a normal physiological response during pregnancy, occurs in approximately 60% to 70% of healthy pregnant women. Nevertheless, it can occasionally be indicative of underlying cardiac or pulmonary conditions. This can pose a challenge for clinicians in determining which patients require further investigation [4]. The physiological changes that occur during pregnancy can affect the cardiovascular and pulmonary systems, leading to dyspnoea or unmasking underlying cardiac pathology [5,7]. With the haemodynamic adaptation that begins in the first trimester, there is an increase in blood volume, leading to an increase in heart rate and stroke volume, alongside a decrease in systemic vascular resistance and blood pressure. These changes lead to an increase in systolic function, cardiac output, preload, left ventricular mass (LVM) and dimensions, while causing a decrease in afterload [4]. In addition, the growing uterus leads to displacement of the diaphragm, mechanical compression of the lungs, and hyperventilation develops in response to a decrease in the functional residual capacity of the lung [3]. Consequently, alterations in the physiology of pregnancy necessitate a specific approach to the assessment of the cardiovascular system. In addition, among the evaluated composite indices, the BNP × uric acid product demonstrated superior discriminative performance compared with conventional biomarkers and showed meaningful associations with echocardiographic markers of right-sided cardiac loading, suggesting that integrating myocardial and metabolic stress pathways may provide a more comprehensive characterisation of dyspnea during late pregnancy.

Recent developments emphasise the need for close, multidisciplinary and continuous monitoring of pregnant women with cardiac problems. In addition, it has been stated that it is important to measure the systolic and diastolic functions, cardiac dimensions and intracardiac pressures of the heart through echocardiography in suspicious cases presenting with dyspnoea [2,7]. A recent study observed a progressive increase in the dimensions of the left and right atria, left ventricular internal diameter (LVID), and right ventricular diameter (RVD) in both systole and diastole, starting from the first trimester and continuing up to the third trimester in normal pregnancy [8]. A study involving 262 peripartum patients with dyspnoea found that 147 of them were diagnosed with physiological dyspnoea, while 11 were diagnosed with peripartum cardiomyopathy (PPCM) [1]. In another study consisting of triplet pregnant women presenting with significant dyspnoea, abnormal echocardiographic findings were detected in 37.5% of the patients [17]. Sharma R et al. observed an increase in LVED pressure and sPAP during pregnancy compared to the pre-pregnancy period [18]. A study by Goland S et al. found that pregnant women experiencing dyspnoea had thicker IVS and higher sPAP when compared to pregnant women without dyspnoea [4]. Mostafavi A et al. found that pregnant women with dyspnoea had significantly higher LVPWd, E/e′ wave ratio, and sPAP compared to the control group [2]. In their study of 32 dyspnoeic patients, McGourty M et al. identified several cardiac abnormalities, including wall motion abnormalities and diastolic dysfunction [5]. Barut MU et al. also demonstrated that even though the LVEDd, LVESd and sPAP of pregnant women with dyspnoea were within normal limits, they were still higher compared to pregnant women without dyspnoea [7]. In our study, the right atrial diameter, right ventricular diameter, and sPAP were all measured to be significantly higher in the dyspnoeic patient group compared to the control group, and sPAP retained the highest diagnostic AUC (0.836) among all echocardiographic parameters. This increase in cardiac chamber dimensions may be a result of the compensation mechanism developed against volume overload during pregnancy [8]. It is also known that high PAP causes dyspnoea [7]. Importantly, the association between the BNP × uric acid product and echocardiographic parameters related to right-sided cardiac loading, including sPAP and right heart dimensions, provides additional biological plausibility for our findings. These observations suggest that elevated combined biomarker burden may parallel subtle hemodynamic alterations despite preserved left ventricular systolic function. From a clinical perspective, these biomarkers are inexpensive, widely available, and rapidly obtainable in emergency department settings. Therefore, BNP-derived uric acid composite indices may serve as adjunctive tools to complement clinical evaluation and echocardiography rather than replace established diagnostic approaches.

Importantly, although these between-group echocardiographic differences were statistically significant, they were small in absolute terms: median sPAP was 15 mmHg in controls versus 21 mmHg in the dyspnoea group, and the right atrial and right ventricular diameters differed by only about 1 mm, with all values remaining within accepted normal limits (sPAP well below the 35–40 mmHg range generally used to suggest pulmonary hypertension). These differences should therefore be interpreted as subtle physiological adaptations rather than as evidence of right ventricular pressure overload or an impending pathological state.

These observations are consistent with more recent work highlighting right-sided involvement in symptomatic pregnancy. In pregnancies complicated by pulmonary hypertension, BNP levels and right ventricular diameter track disease severity and correlate with adverse maternal outcomes [19], and longitudinal echocardiographic studies of pregnant women with pulmonary arterial hypertension have documented progressive increases in sPAP with right ventricular remodelling across the third trimester [20]. Although our cohort excluded overt cardiopulmonary disease, the same right-heart parameters—sPAP and right atrial/ventricular dimensions—were the echocardiographic variables that best distinguished dyspnoeic from asymptomatic women, suggesting that subtle right-sided loading can be detected even when values remain within the physiological range.

There is a growing interest in identifying biochemical markers that can help recognise pregnant women at high risk of adverse cardiovascular events [21]. Natriuretic peptides (NP) may have diagnostic value for worsening existing cardiac failure or newly developing PPCM in pregnant women presenting with acute dyspnoea [22]. Physiological changes during pregnancy can alter the concentrations of NP, making their interpretation challenging as there is not a specific reference value established for pregnancy [9,23,24]. Consistent with the literature, our study demonstrated that BNP levels in the patient group experiencing dyspnoea were significantly elevated compared to the control group, with good discriminative performance (AUC 0.769). Therefore, BNP measurement in pregnant women presenting with dyspnoea may aid in clinical decision-making. Beyond myocardial wall stress, oxidative stress and endothelial dysfunction are increasingly recognised as important contributors to cardiovascular adaptation during pregnancy. Uric acid is not merely a metabolic by-product but also reflects inflammatory activation, oxidative stress, impaired endothelial function, and altered vascular homeostasis. Previous studies have linked elevated uric acid levels with adverse cardiovascular outcomes and pregnancy-related complications. Therefore, combining BNP with uric acid may provide a broader representation of the complex pathophysiological mechanisms underlying dyspnea during late pregnancy. The near-perfect discriminative performance observed for uric acid should be interpreted cautiously and may partly reflect the highly selected study population resulting from the stringent exclusion criteria, the single-centre design, and the relatively homogeneous characteristics of our study cohort. Consequently, the diagnostic performance observed in the present study may not be directly generalizable to broader or more heterogeneous populations, and external validation in independent multicenter cohorts is warranted.

In the non-pregnant emergency setting, natriuretic peptides are used primarily as rule-out tests for acute heart failure. In the multicentre ICON-RELOADED study of patients presenting to the ED with acute dyspnoea, an NT-proBNP concentration below 300 pg/mL excluded acute heart failure with a negative predictive value of approximately 98%, while age-stratified rule-in thresholds (450, 900, and 1800 pg/mL for ages <50, 50–75, and >75 years, respectively) supported its diagnosis [25]. This very high negative predictive value underlies the routine use of NT-proBNP to rapidly exclude acute congestive heart failure in dyspnoeic ED patients. The diagnostic value of BNP itself in acute dyspnoea is likewise well established: in the Breathing Not Properly multinational study, a BNP cut-off of 100 pg/mL identified heart failure with an area under the curve of approximately 0.90, and adding BNP to clinical judgement increased diagnostic accuracy [26,27]. Current heart-failure guidelines continue to recommend rapid natriuretic peptide measurement as a first-line adjunct to distinguish cardiac from non-cardiac dyspnoea in the emergency department [28]. In pregnancy, however, natriuretic peptide concentrations are physiologically altered and pregnancy-specific reference values are lacking [23,24]; consequently, these conventional thresholds cannot be applied directly to the population studied here and require pregnancy-specific recalibration.

Beyond its diagnostic role, the natriuretic peptide axis is also a therapeutic target. Recombinant human BNP (rhBNP; nesiritide), a synthetic form of the endogenous hormone, is approved for acute decompensated heart failure and acts as a vasodilator that lowers cardiac filling pressures and relieves dyspnoea [29]. Because BNP and NT-proBNP concentrations parallel right ventricular dysfunction and haemodynamic severity in pulmonary hypertension [30], rhBNP has been explored as an adjunct in acute decompensated right heart failure across various pulmonary hypertension conditions, with reported reductions in pulmonary and right-heart filling pressures; however, the current evidence base remains limited and largely uncontrolled. These observations are conceptually relevant to our cohort—in which right-sided echocardiographic changes and elevated sPAP were the most prominent haemodynamic findings—but they pertain to the treatment of established disease and should not be extrapolated to the physiological dyspnoea of otherwise healthy pregnancy.

An interesting finding of the present study was that the BNP × uric acid product exhibited better discriminative performance than BNP, D-dimer, and BNP/albumin. Although the exact mechanisms remain speculative, this observation may indicate that the simultaneous assessment of myocardial wall stress and metabolic–oxidative burden captures complementary biological information. The multiplicative interaction between BNP and uric acid may better reflect the complex hemodynamic and metabolic adaptations associated with dyspnea during late pregnancy than isolated biomarkers alone. Nevertheless, because the composite indices are mathematically derived from their individual components, their incremental clinical value beyond uric acid alone should be interpreted cautiously. Accordingly, these findings should be considered hypothesis-generating and require validation in independent multicenter cohorts before BNP-derived composite indices can be recommended for routine clinical triage.

Our composite-index approach is also supported by prognostic data from heart failure cohorts. Uric acid, a marker of oxidative and metabolic stress, carries prognostic information that is largely independent of natriuretic peptides, and combining the two improves risk stratification. Yılmaz Öztekin et al. reported that the combination of uric acid and NT-proBNP predicted adverse outcomes in patients with chronic heart failure more effectively than either marker alone [31], echoing earlier findings in acute heart failure that the joint elevation of uric acid and NT-proBNP identified patients at the highest short-term risk of death or readmission [32]. This incremental value—already demonstrated for hard clinical endpoints in heart failure—provides external biological rationale for our observation that a BNP × uric acid composite outperformed BNP alone in discriminating dyspnoeic pregnant women, and it supports the formal evaluation of such indices against clinical outcomes in future prospective studies.

Beyond BNP in isolation, our study additionally examined serum uric acid and three BNP-derived composite indices, none of which have previously been reported in pregnant patients presenting with dyspnoea. Serum uric acid was markedly elevated in the dyspnoea group and demonstrated an unusually high discriminative performance (AUC 0.998). Uric acid is generated via xanthine oxidase activity during purine catabolism, a pathway upregulated under tissue hypoxia and oxidative stress, and elevated uric acid has previously been linked to adverse outcomes in non-pregnant heart failure populations independent of natriuretic peptide levels [13]. In the non-pregnant cardiovascular literature, ratio-based indices combining uric acid with albumin (the uric acid/albumin ratio) have been proposed as integrative markers of inflammatory and nutritional status with prognostic value in heart failure and acute coronary syndromes [14]. Our findings build on this rationale from a different angle: rather than combining uric acid with albumin, we combined BNP—a marker of myocardial wall stretch—with uric acid and albumin separately, hypothesising that a composite index might better capture the combined haemodynamic and metabolic stress experienced by dyspnoeic pregnant women than any single marker. The BNP × Uric acid product yielded the highest AUC (0.846) among all laboratory markers besides uric acid itself, and exceeded BNP and D-dimer when used individually. This composite index also correlated more strongly with sPAP than BNP alone, raising the possibility that it reflects a combined haemodynamic-metabolic stress signal rather than either dimension alone. By contrast, the BNP/Uric acid ratio performed worse than BNP alone (AUC 0.671), which is mathematically expected: dividing BNP by a denominator that is itself strongly and independently elevated in the patient group partially cancels out, rather than amplifies, the group difference. This pattern suggests that multiplicative rather than ratio-based combination may be the more appropriate way to integrate BNP with a comparably discriminative second marker, whereas ratio-based normalisation may be more useful when the denominator is comparatively stable between groups, as was approximately the case for albumin in our cohort (*p* = 0.846 for albumin alone).

The extremely high AUC observed for uric acid in our cohort (0.998) warrants cautious interpretation. Such near-complete separation between groups is uncommon for a single biomarker in clinical diagnostic studies and could reflect a genuine, strong biological signal, but may also be partly attributable to the relatively narrow, non-overlapping distributions observed in this single-centre cohort, to the deliberately restrictive exclusion criteria (which removed conditions such as pre-eclampsia, renal disease, and hypertension that are themselves associated with altered uric acid metabolism), or to assay or measurement characteristics specific to our laboratory. We therefore present this finding as hypothesis-generating rather than as an immediately generalisable diagnostic threshold, and we emphasise that external validation in independent, multicentre cohorts is required before the uric acid cut-off—or the BNP-derived composite indices built upon it—could be considered for clinical use.

In this context, it must be acknowledged that uric acid measured alone was the single most discriminative marker in our cohort and that none of the composite indices surpassed it. Given the very high—and probably cohort-inflated—AUC of uric acid, the incremental diagnostic value of the BNP-derived indices over uric acid is limited. We therefore present these composite indices not as a means of improving on uric acid for case identification, but as hypothesis-generating constructs that combine two distinct pathophysiological axes—myocardial wall stress (BNP) and metabolic–oxidative stress (uric acid)—whose joint behaviour, and stronger correlation with right-heart echocardiographic parameters, may be biologically informative and warrants testing against clinical endpoints in future studies.

It should also be acknowledged that the composite indices are, by construction, mathematical functions of BNP and uric acid and are therefore strongly correlated with their component markers, as reflected in the correlation analysis. Because uric acid alone already achieved near-complete discrimination in this cohort, the BNP × uric acid product provided no incremental discriminative value over uric acid (DeLong *p* < 0.001 in favour of uric acid), and formal incremental-value analyses such as net reclassification improvement or decision-curve analysis are neither stable nor informative when constructed upon a near-perfect single marker exhibiting quasi-complete separation. The composite indices should therefore be regarded as exploratory constructs that jointly express two complementary pathophysiological axes rather than as statistically superior classifiers. Among them, the BNP/albumin ratio was included as an exploratory normalisation of BNP to a marker of hepatic-synthetic and nutritional status; however, because serum albumin did not differ between the groups, this ratio largely tracked BNP itself and was the least informative of the three indices, whereas the uric-acid–containing indices were more discriminative.

More broadly, it must be emphasised that these indices discriminate the presence of dyspnoea rather than the presence of cardiopulmonary disease per se. Because the great majority of biomarker and echocardiographic values remained within conventional normal ranges, an elevated index should be interpreted as a marker of the symptomatic state and its subtle physiological correlates, not as evidence of a specific cardiopulmonary diagnosis. Given the single-centre design, the absence of external validation, and the lack of confirmed cardiopulmonary diagnoses or postpartum follow-up, these composite indices are best regarded as exploratory markers that require prospective, multicentre validation before any role in emergency assessment could be considered. This caution is consistent with the broader literature on multimodal biochemical evaluation of dyspnoea, in which integrating several complementary markers rather than relying on any single value is advocated to help distinguish physiological adaptation from early cardiopulmonary stress [33].

Despite being a leading cause of maternal mortality, there is currently no suitable indicator available to predict pregnancy-related VTE [12]. The fact that most of the symptoms are also associated with normal pregnancy and the limited use of current diagnostic methods due to radiation exposure makes it difficult to recognise VTE in pregnancy [11]. D-dimer, a marker of thrombosis, is used as a reliable screening tool to rule out VTE in non-pregnant individuals. However, the gradual increase in D-dimer levels throughout pregnancy and the lack of consensus on cut-off values limit its safe use in pregnant women [34]. Hu W et al.’s study suggests that pregnant women with D-dimer levels ≥ 3.70 mg/L may be at risk for VTE, recommending consideration of the D-dimer test [12]. D-dimer levels were significantly higher in dyspnoea patients than in controls in our study (AUC 0.754), suggesting D-dimer remains a useful marker for investigating dyspnoea aetiology in pregnant patients, though in our cohort it was outperformed by uric acid and by the BNP × Uric acid composite index. To the best of our knowledge, this is the first study to investigate BNP-derived uric acid composite indices in third-trimester pregnant women presenting to the emergency department with dyspnea. Nevertheless, these findings should be considered hypothesis-generating and require external validation in larger prospective cohorts before clinical implementation.

### Limitations

The study has several limitations. Firstly, it included a relatively small cohort of participants, specifically pregnant women in their third trimester, and was conducted at a single site. Therefore, the findings should be validated through larger, multicentre studies, and this is particularly important for the BNP/Albumin, BNP/Uric acid, and BNP × Uric acid indices, which are reported here for the first time in this population and require independent confirmation before any proposed cut-off is applied clinically. Secondly, the patient group was only assessed upon admission to the ED; they were discharged once acute conditions were excluded and were not monitored during the postpartum period. Therefore, we do not know how many patients in the dyspnea group were ultimately diagnosed with pathology. Thirdly, as the study included only healthy pregnant women, it is not possible to determine the efficacy of these findings, including the novel composite indices, in detecting any cardiopulmonary disease. The lack of confirmatory diagnosis may limit direct pathological correlation. Despite this, the study’s findings may still provide valuable insight into subclinical hemodynamic and metabolic alterations observed during early evaluation of dyspnoeic pregnancies. Fourthly, due to the emergency setting and patient flow, no validated dyspnea severity instrument, such as the New York Heart Association (NYHA) functional classification or the Borg Dyspnea Scale, was applied. Consequently, correlations between biochemical markers, echocardiographic findings, and standardised measures of symptom severity could not be evaluated. Future studies incorporating validated symptom assessment tools may provide a more comprehensive understanding of the clinical significance of these biomarkers. Fifthly, despite the presence of a statistically significant difference compared to the control group, BNP and echocardiographic findings in the dyspnoea group remained within the normal range. Sixthly, the near-perfect AUC observed for uric acid should be interpreted with caution, as discussed above, given the possibility of cohort-specific or assay-specific factors contributing to this magnitude of separation. Lastly, the control group consisted exclusively of asymptomatic, healthy pregnant women and therefore represented a physiological reference population rather than a clinically relevant differential-diagnostic cohort. Although this design allowed us to characterise biomarker and echocardiographic differences associated with gestational dyspnea, it did not permit direct comparison with pregnant women presenting with confirmed cardiopulmonary diseases. Consequently, part of the observed differences may reflect physiological adaptation to pregnancy rather than disease-specific pathophysiology. Future multicenter studies including both physiological and pathological dyspnea cohorts will be essential to determine the true diagnostic and incremental clinical value of these biomarkers and composite indices. More fundamentally, because the comparison was made against asymptomatic controls rather than against dyspnoeic patients with a confirmed organic cause, and because all major pathological causes of dyspnoea were excluded a priori, the present findings establish only a pathophysiological association between gestational dyspnoea and subtle biomarker and echocardiographic changes. They do not constitute, and should not be interpreted as, a differential-diagnostic or triage algorithm for excluding acute cardiopulmonary emergencies in pregnancy; the higher inflammatory (CRP, WBC) and biomarker levels in the symptomatic group are an expected consequence of comparing symptomatic women with wholly asymptomatic controls. Prospective studies that compare physiological with pathological dyspnoea and include definitive etiological adjudication are therefore required. From a clinical perspective, our results support the use of a combined echocardiographic and biomarker-based approach—potentially incorporating uric acid and the BNP × Uric acid composite index alongside BNP and D-dimer—as an initial triage strategy to identify pregnant patients who may benefit from closer cardiopulmonary evaluation, advanced imaging, or early specialist consultation. In addition, external validation cohorts were not available, and therefore the observed diagnostic performance of the novel BNP-derived uric acid composite indices should be interpreted cautiously. Furthermore, invasive hemodynamic measurements and advanced echocardiographic techniques, such as strain imaging and three-dimensional echocardiography, were not available. Therefore, our findings should be considered hypothesis-generating and require validation in larger multicenter prospective studies before clinical implementation. Accordingly, these biomarkers and composite indices should be regarded as adjunctive research tools rather than standalone diagnostic tests and should always be interpreted in conjunction with clinical assessment, physical examination, echocardiography, and appropriate diagnostic investigations.

In addition, no formal dyspnoea-severity instrument was applied, and detailed acute-presentation data (peripheral oxygen saturation, respiratory rate, chest pain, syncope, suspected pulmonary-embolism work-up, and emergency-department disposition) were not systematically captured; consequently, the biomarker and echocardiographic findings could not be related to an objective measure of symptom burden or to short-term clinical outcomes. This further underscores that the proposed indices are exploratory and hypothesis-generating rather than validated triage tools. Accordingly, these findings should be regarded as hypothesis-generating rather than practice-changing and require confirmation in large multicenter prospective cohorts before clinical implementation.

## 5. Conclusions

In conclusion, dyspnea during late pregnancy may reflect insufficient physiological adaptation to volume overload as well as subtle cardiopulmonary alterations. Although values remained largely within conventional normal ranges, pregnant women presenting to the emergency department with dyspnea exhibited significantly higher levels of D-dimer, BNP, uric acid, right atrial diameter, right ventricular diameter, sPAP, and lateral E/e′ compared with asymptomatic controls. Three novel BNP-derived composite indices—BNP/Albumin, BNP/Uric acid, and BNP × Uric acid—were significantly elevated in the dyspnea group, with the BNP × Uric acid product demonstrating superior diagnostic performance compared with BNP or D-dimer alone. These findings primarily suggest subclinical cardiovascular and metabolic stress rather than overt disease and provide additional reference data for the evaluation of dyspneic pregnant women. In the emergency department setting, conventional biomarkers, echocardiographic parameters, and potentially BNP-derived composite indices should be regarded as markers of subclinical physiological adaptation rather than as a diagnostic or triage tool for acute cardiopulmonary disease; notably, uric acid alone was the strongest discriminator, and the BNP-derived composite indices are offered only as hypothesis-generating constructs. However, these findings should be considered hypothesis-generating and require external validation before clinical implementation.

## 6. Summary Points

- Third-trimester pregnant women presenting to the emergency department with dyspnea exhibited significantly higher levels of BNP, uric acid, D-dimer, right heart dimensions, and sPAP compared with asymptomatic controls.
- Subtle alterations in tissue Doppler parameters and right-sided cardiac dimensions suggest that late-pregnancy dyspnea may be accompanied by mild hemodynamic changes despite preserved left ventricular systolic function.
- Three novel BNP-derived composite indices (BNP/Albumin, BNP/Uric acid, and BNP × Uric acid) were significantly elevated in the dyspnea group.
- Among the evaluated biomarkers, uric acid and the BNP × Uric acid product demonstrated superior discriminative performance, exceeding that of BNP and D-dimer alone.
- BNP-derived uric acid composite indices may provide complementary information beyond conventional biomarkers and echocardiography and may serve as adjunctive tools for early triage and risk assessment in the emergency department setting.

## Figures and Tables

**Table 1:** Demographic Characteristics and Laboratory Findings of Third-Trimester Pregnant Women Presenting with Dyspnea and Asymptomatic Controls.

Variables	Control Group	Patient Group	Overall	*p*-Value
Age, years	27.0 (26.0–31.0)	27.0 (25.0–31.0)	27.0 (25.0–31.0)	0.706 b
BMI, kg/m^2^	29.7 ± 5.3	29.8 ± 5.3	29.8 ± 5.3	0.983 a
Gravidity	2.0 (2.0–3.0)	2.0 (1.0–3.0)	2.0 (1.0–3.0)	0.055 b
Gestational week	33.3 ± 3.9	33.2 ± 3.9	33.2 ± 3.9	0.909 a
MAP, mmHg	84.6 ± 5.2	84.6 ± 5.3	84.6 ± 5.3	0.990 a
Pulse pressure, mmHg	41.0 (39.0–41.0)	41.0 (39.0–41.0)	41.0 (39.0–41.0)	0.879 b
**Laboratory parameters**				
D-dimer (µg/L)	780.0 (680.0–875.0)	1165.0 (805.0–1492.5)	850.0 (705.0–1145.0)	**<0.001 b**
BNP (pg/mL)	19.9 ± 6.7	33.1 ± 16.0	26.0 ± 13.6	**<0.001 a**
Uric acid (mg/dL)	4.9 (4.8–5.0)	5.8 (5.7–6.0)	5.4 (4.9–5.8)	**<0.001 b**
BNP/Albumin	0.6 ± 0.2	0.9 ± 0.5	0.7 ± 0.4	**<0.001 a**
BNP/Uric acid	4.0 ± 1.4	5.6 ± 2.7	4.8 ± 2.2	**<0.001 a**
BNP × Uric acid	97.7 ± 33.0	196.3 ± 97.0	143.6 ± 85.8	**<0.001 a**
Glucose (mg/dL)	86.2 ± 11.6	89.8 ± 16.8	87.9 ± 14.4	0.070 a
Creatinine (mg/dL)	0.5 (0.4–0.5)	0.5 (0.4–0.5)	0.5 (0.4–0.5)	0.856 b
TSH (µUI/mL)	1.9 (1.4–2.6)	1.9 (1.5–2.6)	1.9 (1.4–2.6)	0.750 b
Troponin I (ng/L)	2.5 (2.5–6.0)	2.5 (2.5–6.0)	2.5 (2.5–6.0)	0.448 b
Triglyceride (mg/dL)	152.0 (142.5–162.0)	165.0 (151.2–176.0)	156.0 (145.0–168.0)	**<0.001 b**
CRP (mg/L)	5.0 (3.0–7.0)	8.0 (4.0–12.0)	6.0 (3.0–8.5)	**<0.001 b**
Albumin (g/L)	35.8 ± 2.3	35.9 ± 2.4	35.8 ± 2.4	0.846 a
WBC (10^9^/L)	8.3 ± 1.4	9.8 ± 2.3	9.0 ± 2.0	**<0.001 a**
Hb (g/dL)	11.7 ± 1.1	11.7 ± 1.1	11.7 ± 1.1	0.778 a
PLT (10^9^/L)	249.6 ± 70.7	247.3 ± 70.7	248.5 ± 70.5	0.812 a

BMI: body mass index, MAP: mean artery pressure, BNP: brain natriuretic peptide, TSH: thyroid stimulating hormone, CRP: C-reactive protein, WBC: white blood cell, Hb: haemoglobin, PLT: platelet. a Independent t-test, b Mann–Whitney U test. Significant values (*p* < 0.05) are expressed in bold. The table title has been colored to draw attention.

**Table 2:** Echocardiographic Findings of Third-Trimester Pregnant Women Presenting With Dyspnea and Asymptomatic Controls.

Variables	Control Group	Patient Group	Overall	*p*-Value
LVEDd (mm)	44.0 (42.0–44.0)	44.0 (41.0–44.0)	44.0 (41.5–44.0)	0.727 b
LVESd (mm)	27.0 (27.0–28.0)	27.0 (26.8–28.0)	27.0 (27.0–28.0)	0.672 b
IVSd (mm)	10.0 (10.0–10.0)	10.0 (9.0–10.0)	10.0 (9.0–10.0)	0.677 b
LVPWd (mm)	9.0 (9.0–9.0)	9.0 (8.0–9.0)	9.0 (8.0–9.0)	0.677 b
Left atrium diameter (mm)	28.0 (28.0–28.5)	28.0 (28.0–29.0)	28.0 (28.0–29.0)	0.707 b
Right atrium diameter (mm)	23.0 (22.0–24.0)	24.0 (22.0–25.0)	24.0 (22.0–25.0)	**0.020 b**
Right ventricle diameter (mm)	22.0 (21.0–23.0)	23.0 (22.0–24.0)	23.0 (21.0–24.0)	**0.009 b**
Aortic root diameter (mm)	24.0 (23.0–24.0)	24.0 (23.0–24.0)	24.0 (23.0–24.0)	0.864 b
Aortic systolic diameter (mm)	24.5 (23.4–24.7)	24.5 (23.6–24.7)	24.5 (23.6–24.7)	0.832 b
Aortic diastolic diameter (mm)	18.1 (16.9–19.1)	18.5 (17.3–19.5)	18.3 (17.0–19.3)	0.090 b
sPAP (mmHg)	15.0 (5.0–17.0)	21.0 (17.0–24.2)	17.0 (15.0–21.0)	**<0.001 b**
TAPSE (mm)	26.0 (25.0–26.0)	25.0 (24.0–26.0)	25.0 (25.0–26.0)	**0.024 b**
Mitral E wave velocity (cm/sec)	81.3 ± 10.3	80.4 ± 10.8	80.9 ± 10.5	0.520 a
Mitral A wave velocity (cm/sec)	60.0 ± 9.5	59.8 ± 9.5	59.9 ± 9.5	0.832 a
Septal e’ wave velocity (cm/sec)	10.5 (10.2–11.3)	10.2 (9.5–11.2)	10.3 (10.1–11.2)	**0.002 b**
Septal a’ wave velocity (cm/sec)	7.7 ± 1.1	7.6 ± 1.5	7.6 ± 1.3	0.713 a
Lateral e’ wave velocity (cm/sec)	15.1 (14.9–15.4)	15.0 (13.2–15.3)	15.1 (14.4–15.4)	**<0.001 b**
Lateral a’ wave velocity (cm/sec)	11.6 (10.2–12.2)	11.4 (9.2–12.2)	11.5 (9.8–12.2)	**0.046 b**
E/A wave ratio	1.4 ± 0.1	1.4 ± 0.1	1.4 ± 0.1	0.593 a
Septal E/e′	7.5 ± 1.0	8.0 ± 1.5	7.8 ± 1.3	**0.010 a**
Lateral E/e′	5.3 (4.9–5.8)	5.5 (5.0–6.5)	5.4 (4.9–6.0)	**0.011 b**
Tricuspid regurgitation, Minimal/Mild, n (%)	53 (46.1%)/62 (53.9%)	44 (46.0%)/53 (54.0%)	97 (46.0%)/115 (54.0%)	0.916 c

LVEDd: left ventricle end-diastolic diameter, LVESd: left ventricle end-systolic diameter, IVSd: interventricular septum thickness, LVPWd: left ventricle posterior wall thickness, sPAP: systolic pulmonary artery pressure, TAPSE: tricuspid annular plane systolic excursion. a Independent *t*-test, b Mann–Whitney U test, c Pearson chi-square analysis (3 patients with moderate tricuspid regurgitation, all with sPAP = 32 mmHg, were excluded from this categorical comparison and are reported separately). Left ventricular ejection fraction (LVEF) was 60% for all participants. Significant values (*p* < 0.05) are expressed in bold. The table title has been colored to draw attention.

**Table 3:** Adjusted Differences in Laboratory Parameters Between Dyspneic and Asymptomatic Third-Trimester Pregnant Women.

Variable	Control Group	Patient Group	ANCOVA F	ANCOVA *p*, η^2^p
D-dimer (µg/L) *	780.0 (200.0–1880.0)	1165.0 (200.0–4880.0)	F = 53.19	***p* < 0.001, η^2^p = 0.203**
BNP (pg/mL)	19.9 ± 6.7	33.1 ± 16.0	F = 65.63	***p* < 0.001, η^2^p = 0.239**
Uric acid (mg/dL) *	4.9 (4.4–5.6)	5.8 (5.5–6.9)	F = 1221.81	***p* < 0.001, η^2^p = 0.854**
BNP/Albumin	0.6 ± 0.2	0.9 ± 0.5	F = 62.31	***p* < 0.001, η^2^p = 0.230**
BNP/Uric acid	4.0 ± 1.4	5.6 ± 2.7	F = 29.05	***p* < 0.001, η^2^p = 0.122**
BNP × Uric acid *	97.7 ± 33.0	196.3 ± 97.0	F = 101.07	***p* < 0.001, η^2^p = 0.326**
Triglyceride (mg/dL)	152.0 (114.0–180.0)	165.0 (128.0–246.0)	F = 35.82	***p* < 0.001, η^2^p = 0.146**
CRP (mg/L) *	5.0 (2.0–13.0)	8.0 (1.9–37.0)	F = 26.62	***p* < 0.001, η^2^p = 0.113**
WBC (10^9^/L)	8.3 ± 1.4	9.8 ± 2.3	F = 38.71	***p* < 0.001, η^2^p = 0.156**

* Logarithmic transformation was performed to convert skewed variables into a normal distribution pattern prior to ANCOVA modelling. Covariates used for adjustment in the model: age, BMI, MAP, and gestational week. η^2^p: partial eta-squared. BNP: brain natriuretic peptide, CRP: C-reactive protein, WBC: white blood cell. Significant values (*p* < 0.05) are expressed in bold. The table title has been colored to draw attention.

**Table 4:** Diagnostic Performance of Laboratory and Echocardiographic Parameters for Identifying Dyspnea in Third-Trimester Pregnant Women.

Variable	AUC	95% CI	Cut-Off	Sensitivity (%)	Specificity (%)	*p*
**Laboratory parameters**						
D-dimer (µg/L)	**0.754**	0.689–0.820	≥1090.00	54.0	95.7	<0.001
BNP (pg/mL)	**0.769**	0.705–0.833	≥25.30	65.0	82.6	<0.001
Uric acid (mg/dL)	**0.998**	0.991–1.000	≥5.60	99.0	97.4	<0.001
BNP/Albumin	**0.765**	0.700–0.829	≥0.74	63.0	86.1	<0.001
BNP/Uric acid	**0.671**	0.598–0.743	≥5.72	46.0	89.6	<0.001
BNP × Uric acid	**0.846**	0.792–0.900	≥138.00	70.0	91.3	<0.001
Triglyceride (mg/dL)	**0.704**	0.634–0.774	≥164.00	55.0	78.3	<0.001
CRP (mg/L)	**0.680**	0.608–0.752	≥7.30	53.0	80.9	<0.001
WBC (10^9^/L)	**0.705**	0.634–0.775	≥9.16	58.0	75.7	<0.001
**Echocardiographic parameters**						
Right atrium diameter (mm)	**0.591**	0.514–0.667	≥25.00	37.0	79.1	0.020
Right ventricle diameter (mm)	**0.601**	0.525–0.677	≥24.00	38.0	79.1	0.009
sPAP (mmHg)	**0.836**	0.780–0.891	≥18.00	74.0	80.0	<0.001
Septal e′ wave velocity (cm/sec)	**0.622**	0.547–0.698	≤9.50	27.0	100.0	0.001
Lateral e′ wave velocity (cm/sec)	**0.636**	0.561–0.710	≤13.70	35.0	98.3	<0.001
Lateral a′ wave velocity (cm/sec)	**0.579**	0.502–0.656	≤9.50	33.0	89.6	0.044
Lateral E/e′	**0.601**	0.525–0.677	≥5.90	37.0	83.5	0.009

ROC: Receiver Operating Characteristic, AUC: area under the curve, CI: Confidence Interval, BNP: brain natriuretic peptide, CRP: C-reactive protein, WBC: white blood cell, sPAP: systolic pulmonary artery pressure. Cut-off values determined based on the Youden J index. For septal e′, lateral e′, and lateral a′ wave velocities, lower values are associated with the dyspnoea (positive) group. 95% CI for uric acid truncated at the analytic upper bound of 1.000. The table title has been colored to draw attention.

**Table 5:** Correlations Between Demographic Variables and Laboratory Parameters, Including Novel BNP-Derived Uric Acid Composite Indices.

	Age	BMI	MAP	D-Dimer (µg/mL)	BNP (pg/mL)
**Age**	–	0.236	0.879	−0.029	0.080
*p*	–	**<0.001**	**<0.001**	0.674	0.243
**BMI**	0.236	–	0.251	0.035	−0.057
*p*	**<0.001**	–	**<0.001**	0.609	0.404
**MAP**	0.879	0.251	–	−0.054	0.153
*p*	**<0.001**	**<0.001**	–	0.434	**0.025**
**D-dimer (µg/mL)**	−0.029	0.035	−0.054	–	0.481
*p*	0.674	0.609	0.434	–	**<0.001**
**BNP (pg/mL)**	0.080	−0.057	0.153	0.481	–
*p*	0.243	0.404	**0.025**	**<0.001**	–
**Uric acid (mg/dL)**	0.154	0.134	0.115	0.395	0.404
*p*	**0.024**	**0.050**	0.093	**<0.001**	**<0.001**
**BNP/Albumin**	0.095	−0.045	0.173	0.487	0.987
*p*	0.167	0.516	**0.011**	**<0.001**	**<0.001**
**BNP/Uric acid**	0.037	−0.089	0.117	0.429	0.971
*p*	0.593	0.194	0.088	**<0.001**	**<0.001**
**BNP × Uric acid**	0.109	−0.026	0.176	0.492	0.979
*p*	0.110	0.709	**0.010**	**<0.001**	**<0.001**

Spearman correlation analysis. rho (ρ): correlation coefficient, *p*: significance value. BMI: body mass index, MAP: mean artery pressure, BNP: brain natriuretic peptide. Significant values (*p* < 0.05) are expressed in bold. The table title has been colored to draw attention.

**Table 6:** Correlation Matrix of Biomarkers and Echocardiographic Parameters in Third-Trimester Pregnant Women.

	Age	BMI	MAP	D-Dimer	BNP	Uric Acid	BNP × UA
**LVEDd (mm)**	0.232	0.292	0.257	−0.008	0.007	0.051	0.017
*p*	**<0.001**	**<0.001**	**<0.001**	0.911	0.920	0.458	0.802
**LVESd (mm)**	0.342	0.220	0.352	−0.062	−0.004	0.039	0.009
*p*	**<0.001**	**0.001**	**<0.001**	0.368	0.958	0.567	0.892
**IVSd (mm)**	0.287	0.366	0.242	−0.019	−0.121	0.039	−0.100
*p*	**<0.001**	**<0.001**	**<0.001**	0.777	0.076	0.566	0.144
**LVPWd (mm)**	0.290	0.364	0.246	−0.005	−0.120	0.043	−0.099
*p*	**<0.001**	**<0.001**	**<0.001**	0.944	0.078	0.528	0.149
**Left atrium diameter (mm)**	0.186	0.150	0.148	−0.016	0.030	0.067	0.029
*p*	**0.006**	**0.028**	**0.030**	0.810	0.657	0.329	0.671
**Right atrium diameter (mm)**	0.509	0.278	0.512	0.043	0.172	0.223	0.206
*p*	**<0.001**	**<0.001**	**<0.001**	0.533	**0.011**	**<0.001**	**0.002**
**Right ventricle diameter (mm)**	0.490	0.279	0.495	0.056	0.179	0.228	0.213
*p*	**<0.001**	**<0.001**	**<0.001**	0.411	**0.009**	**<0.001**	**0.002**
**Aortic root diameter (mm)**	0.256	0.167	0.214	−0.114	−0.041	0.038	−0.033
*p*	**<0.001**	**0.015**	**0.002**	0.096	0.548	0.581	0.631
**Aortic systolic diameter (mm)**	0.225	0.107	0.159	−0.100	−0.030	0.035	−0.024
*p*	**<0.001**	0.118	**0.020**	0.144	0.664	0.615	0.725
**Aortic diastolic diameter (mm)**	0.508	0.222	0.463	−0.053	0.028	0.182	0.063
*p*	**<0.001**	**0.001**	**<0.001**	0.437	0.685	**0.007**	0.356
**sPAP (mmHg)**	0.298	0.012	0.318	0.201	0.398	0.508	0.466
*p*	**<0.001**	0.866	**<0.001**	**0.003**	**<0.001**	**<0.001**	**<0.001**
**TAPSE (mm)**	−0.460	−0.286	−0.438	−0.033	−0.085	−0.268	−0.135
*p*	**<0.001**	**<0.001**	**<0.001**	0.626	0.214	**<0.001**	**0.048**
**Mitral E wave velocity (cm/sec)**	0.005	0.034	0.008	−0.256	−0.022	−0.061	−0.027
*p*	0.947	0.624	0.907	**<0.001**	0.747	0.374	0.698
**Mitral A wave velocity (cm/sec)**	0.100	0.009	0.094	−0.146	0.093	0.022	0.091
*p*	0.145	0.898	0.172	**0.032**	0.172	0.749	0.183
**Septal e′ wave velocity (cm/sec)**	−0.140	−0.115	−0.130	−0.191	−0.114	−0.255	−0.141
*p*	**0.040**	0.094	0.056	**0.005**	0.094	**<0.001**	**0.039**
**Septal a′ wave velocity (cm/sec)**	0.022	−0.212	0.022	−0.184	−0.077	−0.092	−0.081
*p*	0.750	**0.002**	0.750	**0.007**	0.262	0.180	0.238
**Lateral e′ wave velocity (cm/sec)**	−0.190	−0.179	−0.144	−0.082	−0.024	−0.299	−0.072
*p*	**0.005**	**0.008**	**0.034**	0.233	0.724	**<0.001**	0.293
**Lateral a′ wave velocity (cm/sec)**	−0.078	−0.142	−0.045	−0.029	0.001	−0.161	−0.021
*p*	0.254	**0.038**	0.512	0.667	0.991	**0.018**	0.762
**E/A wave ratio**	−0.115	−0.006	−0.114	−0.059	−0.145	−0.101	−0.147
*p*	0.093	0.930	0.097	0.393	**0.034**	0.139	**0.031**
**Septal E/e′**	0.106	0.113	0.112	−0.070	0.053	0.144	0.073
*p*	0.122	0.098	0.102	0.308	0.442	**0.035**	0.284
**Lateral E/e′**	0.108	0.102	0.094	−0.156	0.010	0.189	0.044
*p*	0.113	0.138	0.171	**0.022**	0.879	**0.006**	0.519

Spearman correlation analysis. rho (ρ): correlation coefficient, *p*: significance value. BMI: body mass index, MAP: mean artery pressure, BNP: brain natriuretic peptide, UA: uric acid, LVEDd: left ventricle end-diastolic diameter, LVESd: left ventricle end-systolic diameter, IVSd: interventricular septum thickness, LVPWd: left ventricle posterior wall thickness, sPAP: systolic pulmonary artery pressure, TAPSE: tricuspid annular plane systolic excursion. Significant values (*p* < 0.05) are expressed in bold. The table title has been colored to draw attention.

## Data Availability

If the data is requested by the journal, we can share it in the additional information file.

## Abbreviations

A′	Late diastolic mitral annular velocity
AUC	Area under the curve
ASE	American Society of Echocardiography
BMI	Body mass index
BNP	B-type natriuretic peptide
CI	Confidence interval
COPD	Chronic obstructive pulmonary disease
CRP	C-reactive protein
DVT	Deep vein thrombosis
E′	Early diastolic mitral annular velocity
ED	Emergency department
HF	Heart failure
IVSd	Interventricular septum thickness
IQR	Interquartile range
LVEDd	Left ventricular end-diastolic diameter
LVESd	Left ventricular end-systolic diameter
LVEF	Left ventricular ejection fraction
LVID	Left ventricular internal diameter
LVPWd	Left ventricular posterior wall thickness
MAP	Mean arterial pressure
NP	Natriuretic peptide
NT-proBNP	N-terminal pro-B-type natriuretic peptide
PPCM	Peripartum cardiomyopathy
PE	Pulmonary embolism
PLT	Platelet count
ROC	Receiver operating characteristic
RVD	Right ventricular diameter
sPAP	Systolic pulmonary artery pressure
TAPSE	Tricuspid annular plane systolic excursion
TSH	Thyroid-stimulating hormone
UA	Uric acid
VTE	Venous thromboembolism
WBC	White blood cell count
η^2^p	Partial eta-squared

## References

[1] Wang W.W., Wang Y. (2018). Peripartum women with dyspnea in the emergency department: Is it peripartum cardiomyopathy?. Medicine.

[2] Mostafavi A., Feizian M., Fotook Kiaei S.Z., Tabatabaei S.A. (2022). Dyspnea in pregnancy might be related to the incomplete physiological adaptation of the heart. J. Cardiovasc. Thorac. Res..

[3] Lee S.Y., Chien D.K., Huang C.H., Shih S.C., Lee W.C., Chang W.H. (2017). Dyspnea in pregnancy. Taiwan J. Obstet. Gynecol..

[4] Goland S., Perelman S., Asalih N., Shimoni S., Walfish O., Hallak M., Hagay Z., George J., Shotan A., Blondheim D.S. (2015). Shortness of breath during pregnancy: Could a cardiac factor be involved?. Clin. Cardiol..

[5] McGourty M., Skaritanov E., Kovell L., Wilkie G. (2024). Cardiac evaluation in pregnant patients with dyspnea and palpitations. Am. J. Obstet. Gynecol. MFM.

[6] Afari H.A., Davis E.F., Sarma A.A. (2021). Echocardiography for the pregnant heart. Curr. Treat. Options Cardiovasc. Med..

[7] Barut M.U., Güngören F., Kaçmaz C. (2019). Assessment of clinical and echocardiographic findings of pregnant women with dyspnea. Med. Sci. Monit..

[8] Iloeje U.N., Mankwe A.C., Kweki A.G., Aiwuyo H.O., Oladimeji O.M., Emenena I., Akpa M.R., Odia O.J., Jesuorobo D.E. (2023). Cardiac dimensions in normal pregnancy: A prospective study. Cureus.

[9] Dos Santos F., Dennehy N., Steer P.J., Johnson M.R. (2023). B-type natriuretic peptide in low-risk pregnancy and pregnancy with congenital heart disease. Int. J. Gynaecol. Obstet..

[10] Malhamé I.M., Hurlburt H., Larson L., Poppas A., Nau C., Bourjeily G., Mehta N. (2019). Sensitivity and specificity of B-type natriuretic peptide in diagnosing heart failure in pregnancy. Obstet. Gynecol..

[11] Gutiérrez García I., Pérez Cañadas P., Martínez Uriarte J., García Izquierdo O., Angeles Jódar Pérez M., García de Guadiana Romualdo L. (2018). D-dimer during pregnancy: Establishing trimester specific reference intervals. Scand. J. Clin. Lab. Investig..

[12] Hu W., Wang Y., Li J., Huang J., Pu Y., Jiang Y., Xu D., Ding Z., Zhao B., Luo Q. (2020). The predictive value of d-dimer test for venous thromboembolism during puerperium: A prospective cohort study. Clin. Appl. Thromb. Hemost..

[13] Qin S., Xiang M., Gao L., Cheng X., Zhang D. (2024). Uric acid is a biomarker for heart failure, but not therapeutic target: Result from a comprehensive meta-analysis. ESC Heart Fail..

[14] Liu X., Chu A., Ding X. (2024). Elevated uric acid to serum albumin ratio: A predictor of short-term outcomes in Chinese heart failure patients. Front. Nutr..

[15] Lang R.M., Badano L.P., Mor-Avi V., Afilalo J., Armstrong A., Ernande L., Flachskampf F.A., Foster E., Goldstein S.A., Kuznetsova T. (2015). Recommendations for Cardiac Chamber Quantification by Echocardiography in Adults: An Update from the American Society of Echocardiography and the European Association of Cardiovascular Imaging. J. Am. Soc. Echocardiogr..

[16] DeLong E.R., DeLong D.M., Clarke-Pearson D.L. (1988). Comparing the areas under two or more correlated receiver operating characteristic curves: A nonparametric approach. Biometrics.

[17] Elhenicky M., Distelmaier K., Mailath-Pokorny M., Worda C., Langer M., Worda K. (2016). Abnormal maternal echocardiographic findings in triplet pregnancies presenting with dyspnoea. Wien. Klin. Wochenschr..

[18] Sharma R., Kumar A., Aneja G.K. (2016). Serial changes in pulmonary hemodynamics during pregnancy: A non-invasive study using doppler echocardiography. Cardiol. Res..

[19] Chen G., Zhang Z., Zhao X., Huang K. (2025). Maternal and neonatal outcomes in pregnancies complicated with pulmonary hypertension: A retrospective cohort study. Eur. J. Med. Res..

[20] Olsson K.M., Fuge J., Park D.-H., Kamp J.C., Berliner D., von Kaisenberg C., Hoeper M.M. (2024). Right heart function during and after pregnancy in women with pulmonary arterial hypertension. ERJ Open Res..

[21] Sarma A.A., Scott N.S. (2023). Dynamics of NT-proBNP in pregnancy: Why values may be elevated in the first trimester. JACC Adv..

[22] Burlingame J.M., Yamasato K., Ahn H.J., Seto T., Tang W.H.W. (2017). B-type natriuretic peptide and echocardiography reflect volume changes during pregnancy. J. Perinat. Med..

[23] Dockree S., Brook J., Shine B., James T., Vatish M. (2021). Pregnancy-specific reference intervals for BNP and NT-pro BNP—Changes in natriuretic peptides related to pregnancy. J. Endocr. Soc..

[24] Minhas A.S., Rooney M.R., Fang M., Zhang S., Ndumele C.E., Tang O., Schulman S.P., Michos E.D., McEvoy J.W., Echouffo-Tcheugui J.B. (2023). Prevalence and correlates of elevated NT-proBNP in pregnant women in the general U.S. population. JACC Adv..

[25] Januzzi J.L., Chen-Tournoux A.A., Christenson R.H., Doros G., Hollander J.E., Levy P.D., Nagurney J.T., Nowak R.M., Pang P.S., Patel D. (2018). N-terminal pro-B-type natriuretic peptide in the emergency department: The ICON-RELOADED study. J. Am. Coll. Cardiol..

[26] Maisel A.S., Krishnaswamy P., Nowak R.M., McCord J., Hollander J.E., Duc P., Omland T., Storrow A.B., Abraham W.T., Wu A.H. (2002). Rapid measurement of B-type natriuretic peptide in the emergency diagnosis of heart failure. N. Engl. J. Med..

[27] McCullough P.A., Nowak R.M., McCord J., Hollander J.E., Herrmann H.C., Steg P.G., Duc P., Westheim A., Omland T., Knudsen C.W. (2002). B-type natriuretic peptide and clinical judgment in emergency diagnosis of heart failure: Analysis from Breathing Not Properly (BNP) Multinational Study. Circulation.

[28] McDonagh T.A., Metra M., Adamo M., Gardner R.S., Baumbach A., Böhm M., Burri H., Butler J., Čelutkienė J., Chioncel O. (2021). 2021 ESC guidelines for the diagnosis and treatment of acute and chronic heart failure. Eur. Heart J..

[29] Publication Committee for the VMAC Investigators (2002). Intravenous nesiritide vs nitroglycerin for treatment of decompensated congestive heart failure: A randomized controlled trial. JAMA.

[30] Nagaya N., Nishikimi T., Uematsu M., Satoh T., Kyotani S., Sakamaki F., Kakishita M., Fukushima K., Okano Y., Nakanishi N. (2000). Plasma brain natriuretic peptide as a prognostic indicator in patients with primary pulmonary hypertension. Circulation.

[31] Yılmaz Öztekin G.M., Genç A., Çağırcı G., Arslan Ş. (2022). Prognostic value of the combination of uric acid and NT-proBNP in patients with chronic heart failure. Hell. J. Cardiol..

[32] Park H.-S., Kim H., Sohn J.-H., Shin H.-W., Cho Y.-K., Yoon H.-J., Nam C.-W., Hur S.-H., Kim Y.-N., Kim K.-B. (2010). Combination of uric acid and NT-proBNP: A more useful prognostic marker for short-term clinical outcomes in patients with acute heart failure. Korean J. Intern. Med..

[33] Tetaj N., Segreti A., Piccirillo F., Pelullo M., Crispino S.P., Ciancio M., Ussia G.P., Grigioni F. (2026). Diagnostic and prognostic value of arterial blood gas and electrolyte analyses in heart failure. Rev. Cardiovasc. Med..

[34] Ker J.A., Soma-Pillay P. (2018). NT-proBNP: When is it useful in obstetric medicine?. Obstet. Med..

